# Evaluating Ocular Symptoms and Tear Film Cytokine Profiles in Symptomatic COVID-19 Patients

**DOI:** 10.3390/jcm11092647

**Published:** 2022-05-08

**Authors:** Anna Niedźwiedź, Miłosz Kawa, Ewa Pius-Sadowska, Agnieszka Kuligowska, Alicja Ziontkowska, Dawid Wrzałek, Miłosz Parczewski, Krzysztof Safranow, Krzysztof Kozłowski, Bogusław Machaliński, Anna Machalińska

**Affiliations:** 1 Department of General Pathology, Pomeranian Medical University, Al. Powstańców Wielkopolskich 72, 70-111 Szczecin, Poland; ania.niedzwiedz@gmail.com (A.N.); kawamilosz@gmail.com (M.K.); ewapius@wp.pl (E.P.-S.); boguslaw.machalinski@pum.edu.pl (B.M.); 2 First Department of Ophthalmology, Pomeranian Medical University, Al. Powstańców Wielkopolskich 72, 70-111 Szczecin, Poland; agnieszka.kaleta91@gmail.com (A.K.); alicjaziontkowska@gmail.com (A.Z.); dawidwrzalek@me.com (D.W.); 3 Department of Infectious, Tropical Diseases and Immune Deficiency, Pomeranian Medical University, Arkońska 4 Street, 71-455 Szczecin, Poland; mparczewski@yahoo.co.uk; 4 Department of Biochemistry and Medical Chemistry, Pomeranian Medical University, Al. Powstańców Wielkopolskich 72, 70-111 Szczecin, Poland; chrissaf@mp.pl; 5 Department of Constitutional Law, Faculty of Law and Administration, Jagiellonian University, Bracka 12 Street, 31-005 Krakow, Poland; krzys.kozlowski@uj.edu.pl

**Keywords:** COVID-19, ocular symptoms, Luminex, tear film cytokine levels

## Abstract

Background: This study investigated the presence and duration of ophthalmic symptoms in the early phase of COVID-19 to assess the corresponding local immune response on the ocular surface. Methods: The study included data from 180 COVID-19 patients and 160 age-matched healthy controls. The main finding was the occurrence of ophthalmological manifestations at the time of admission to the hospital and during the preceding 7 days. Tear film concentrations of TNF-α, IL-1b, IL-2, IL-4, IL-5, IL-6, IL-8, IL-10, IL-12 p70, GM-CSF, and IFN-γ were determined by a magnetic bead assay. Results: Among the COVID-19 patients, 12.64% had at least one ocular symptom at the time of admission, and 24.14% had symptoms within the preceding 7 days (*p* < 0.001 vs. controls). We found that the COVID-19 patients complained more frequently about eye tearing (*p* = 0.04) and eye pain (*p* = 0.01) than controls. A multivariate analysis of the patients and controls adjusted for age and sex revealed that COVID-19 was an independent factor associated with higher VEGF and IL-10 tear film concentrations (β = +0.13, *p* = 0.047 and β = +0.34, *p* < 0.001, respectively) and lower IL-1β, IL-8, and GM-CSF levels (β = −0.25, *p* < 0.001; β = −0.18, *p* = 0.004; and β = −0.82, *p* = 0.0 respectively). Conclusions: SARS-CoV-2 does not attract a strong local response of the conjunctival immune system; therefore, ophthalmic symptoms may not constitute a substantial element in the clinical picture of novel COVID-19 infection.

## 1. Introduction

SARS-CoV-2 was first detected in December 2019 in the Chinese city of Wuhan and is known to cause coronavirus disease 2019 (COVID-19). On 11 March 2020, the World Health Organization declared COVID-19 a global pandemic. Due to the fact that ocular symptoms have also been reported in COVID-19 patients, some clinicians have expressed concerns about the transmission of SARS-CoV-2 via the tears and conjunctival secretions of infected patients [1,2]. Moreover, several affected subjects presented first with conjunctivitis before the onset of respiratory tract symptoms, implying that the ocular route could be a probable transmission path of SARS-CoV-2 to the rest of the human body under specific conditions [3,4].

Since the ocular surface could serve as a potential point of entry for SARS-CoV-2, this route of novel coronavirus transmission is under investigation.

There is only one report on the detection of pro- and anti-inflammatory components in tears and conjunctival fluid from COVID-19 patients. However, that study comprised only 62 COVID-19 patients and 41 corresponding control individuals, which is a relatively small group with which to reliably correlate the molecular results with ocular surface symptoms [5].

Therefore, the aim of our study was to estimate the presence of ocular symptoms in SARS-CoV-2-positive patients manifesting classical COVID-19 symptoms and to compare it with that in SARS-CoV-2-negative controls. In addition, we evaluated the levels of selected proinflammatory cytokines and growth factors in tears collected from both groups recruited to the study. To our knowledge, comprehensive data on COVID-19-related ophthalmological symptoms (higher prevalence of eye itching, burning, tearing, redness, and pain of the eye) among patients with symptomatic COVID-19 and their correspondence with the inflammatory state in tears have not been previously reported.

## 2. Materials and Methods

### 2.1. Study Group

This retrospective cohort study included 180 patients diagnosed with COVID-19 in the Department of Infectious, Tropical Diseases and Immune Deficiency of Pomeranian Medical University in Szczecin, Poland, based on positive RT-polymerase chain reaction (RT–PCR) results for SARS-CoV-2 obtained from nasopharyngeal swabs. Only COVID-19 symptomatic patients were included in the study. The control group consisted of 160 age-matched healthy individuals with negative RT–PCR results for SARS-CoV-2 obtained from nasopharyngeal swabs and negative ELISA results for SARS-CoV-2-specific IgG, IgM, and IgA antibodies that were recruited from among the hospital staff. Both groups enrolled in the study underwent tear sampling and were asked to complete detailed questionnaires regarding their general and ophthalmologic health. An informed consent form in accordance with the tenets of the Declaration of Helsinki was signed by all participants before study enrolment.

### 2.2. General Health and Ophthalmological Questionnaire

A general health questionnaire to assess the presence of the most common chronic diseases and related medications taken was administered. The survey collected data on the presence of diabetes mellitus, hypertension, ischemic heart disease and past heart attacks, liver and renal disorders, rheumatic joint disease, and neoplasms. All participants were asked whether they used antihypertensive and other cardiac drugs, statins, glucocorticoids, immunosuppressant and anti-allergic drugs, anticoagulants, and NSAIDs.

An ophthalmologic questionnaire to assess the presence of ocular symptoms and their duration at the time of enrolment and in the 7 days preceding admission to the hospital was filled in by the participants. In addition, the survey incorporated the subjects’ current and past ophthalmic disorder history, including contact lens use, any past anterior eye injuries or eye surgeries, allergic seasonal conjunctivitis, and dry eye syndrome. Eye-COVID scores (ECSs) were marked as 0 (absent) or 1 (present) for the following parameters: (i) swelling of the eyelids, (ii) itchy eyes, (iii) eye burning, (iv) excessive tearing, (v) redness of the eye, (vi) feeling of sand under the eyelids, (vii) presence of discharge, (viii) sticking of the eyelids, (ix) photophobia, (x) feeling of stiffness in the eyeball, (xi) eye pain, (xii) visual impairment, (xiii) cloudy vision, and (xiv) blurry vision.

### 2.3. Tear Sample Collection and Analysis

To assess the expression of proinflammatory cytokines in the tear film, a tear sample was collected from each study participant. Schirmer’s strips (TearFloTM, HUB Pharmaceuticals, Scottsdale, AZ, USA) were used to collect the tear fluid in all subjects. The strip was placed in the lower conjunctival sac, and tears were allowed to diffuse into the strip until reaching 20 mm on the strip scale. The subjects were allowed to blink freely during this time. The Schirmer strip was then placed into an Eppendorf tube and frozen at −80 °C. Tear fluid was extracted from the Schirmer strips by agitating small cut pieces of these strips in 300 μL of phosphate buffered saline (PBS) solution in a sterile 1.5 mL microcentrifuge tube at 4 °C for 3 h. Tear fluid was then eluted by centrifugation for 10 min and stored at −80 °C until further use. The concentrations of TNF-α, IL-1b, IL-2, IL-4, IL-5, IL-6, IL-8, IL-10, IL-12 p70, GM-CSF, VEGF, and IFN-γ in tear fluids were measured by multiplex fluorescent bead-based immunoassays (Luminex Corporation, Austin, TX, USA) using commercial R&D Systems Luminex Performance Human High Sensitivity Cytokine Magnetic Panel A (R&D Systems, Minneapolis, MN, USA). Next, 100-microliter aliquots of each standard, control and sample, were added to the plate together with 25 µL of multiplex antibody capture microparticle solution, and the plate was incubated with agitation for 3 h at room temperature. Subsequently, each well was washed with 100 µL of wash buffer three times using a hand-held magnet. A total of fifty microliters of detection antibody cocktail was pipetted into each well, and the plate was sealed and incubated at room temperature for 1 h on a shaker. After this step, the wash was repeated, and 50 µL of streptavidin–phycoerythrin mixture was added to the plate and incubated with agitation for 30 min in the dark. Finally, after washing, the microspheres in each well were resuspended in 100 µL of wash buffer and shaken at room temperature for 5 min. The plate was then read and analyzed on a Luminex 200 analyzer, and analyte concentrations were determined from five different standard curves showing MFI (median fluorescence intensity) vs. protein concentration.

### 2.4. Statistical Analysis

The statistical analysis was conducted using Statistica 13.3 software. The Mann–Whitney U test was used to compare quantitative and rank variables between groups. The strength of associations between quantitative and rank variables was measured with Spearman rank correlation coefficient (Rs). Fisher’s exact test was used to compare qualitative variables between groups. Quantitative variables were summarized with the mean ± standard deviation (SD) and/or median (interquartile range (IQR)). Multivariate analysis adjusted for age and sex was performed using general linear model (GLM) with logarithmically transformed cytokine concentrations in tears as dependent variables to determine whether SARS-CoV-2 is an independent factor associated with these concentrations. A *p* value of less than 0.05 was considered significant.

## 3. Results

### 3.1. Clinical Data

The characteristics of the patients with positive and negative PCR results for SARS-CoV-2 are shown in Table 1. The BMI values were reported to be significantly higher in the SARS-CoV-2-positive group than in the control group (*p* < 0.001). Among the patients with symptomatic COVID-19, there was also a significantly higher prevalence of hypertension, diabetes mellitus, and ischemic heart disease, whereas rheumatic joint disease was reported to be more frequent among the SARS-CoV-2-negative subjects (*p* < 0.001, *p* < 0.001, *p* = 0.03, and *p* = 0.001, respectively). Regarding the data on medication use, the SARS-CoV-2-positive patients reported the use of statins, antihypertensive drugs, and other cardiac drugs more frequently than the controls (*p* = 0.01, *p* < 0.001 and *p* = 0.004, respectively).

### 3.2. Ophthalmological Data

The ophthalmological characteristics of the patients with positive and negative SARS-CoV-2 PCR results are shown in Table 2. Among the COVID-19 patients, 12.64% had at least one ECS at the time of examination in the admission department and 0.66% of the controls had at least one ECS (*p* < 0.001). We also found a higher prevalence of ocular symptoms in the COVID-19 group within the 7 days preceding the examination at the admission department (24.14% of SARS-CoV-2-positive patients vs. 1.32% of negative controls; *p* < 0.001). When analyzing specific ocular symptoms, we found that the COVID-19 patients complained more frequently about eye tearing and eye pain than the controls (*p* = 0.04 and *p* = 0.01, respectively). Consequently, when analyzing the eye symptoms during the 7-day period before admission to the hospital ward, it was found that the symptomatic SARS-CoV-2-positive patients reported a higher prevalence of eye itching, burning, tearing, redness, and pain in the eye (*p* = 0.01, *p* = 0.01, *p* = 0.001, *p* = 0.02, and *p* < 0.001, respectively). In addition, the COVID-19 patients also reported light sensitivity and visual impairment more frequently (*p* = 0.04 and *p* = 0.03, respectively).

In addition, to evaluate the potential impact of concomitant ocular diseases, medication use, and past ocular surgeries on present ocular symptoms, we analyzed the data regarding the ophthalmic history between the groups. We found no differences between the compared groups in terms of the presence of allergic conjunctivitis (3.45% of COVID-19 patients vs. 3.29% of SARS-CoV-2-negative controls; *p* = 1.00), dry eye (3.45% of COVID-19 patients vs. 3.29% of SARS-CoV-2-negative controls; *p* = 1.00), or infectious conjunctivitis (2.30% of COVID-19 patients vs. 5.92% of SARS-CoV-2-negative controls; *p* = 0.15) within one year preceding the COVID-19 event. Similarly, we found no differences between the groups with regard to contact lens use (5.75% of COVID-19 patients vs. 5.92% of SARS-CoV-2-negative controls; *p* = 1.00), past ocular injuries within the previous 12 months (1.15% of COVID-19 patients vs. 0.66% of SARS-CoV-2-negative controls; *p* = 1.00), ocular surgeries within the previous 12 months (1.15% of COVID-19 patients vs. 1.32% of SARS-CoV-2-negative controls; *p* = 1.00), or the therapeutic use of drops for dry-eye syndrome (7.47% of COVID-19 patients vs. 10.53% of SARS-CoV-2-negative controls; *p* = 0.44) and glaucoma (2.84% of COVID-19 patients vs. 1.32% of SARS-CoV-2-negative controls; *p* = 0.46).

### 3.3. Levels of Selected Cytokines in Tears of Patients with COVID-19

**Figure 1:**
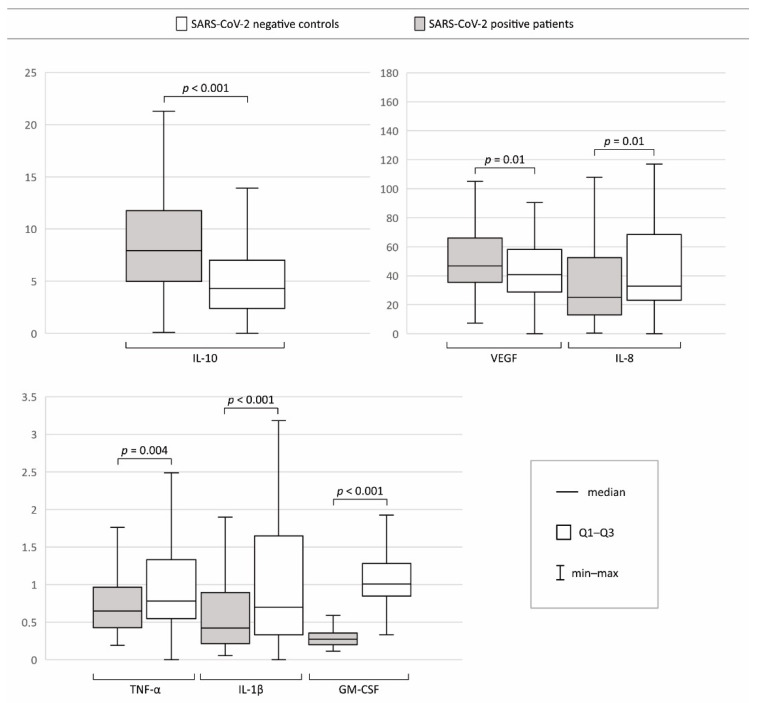
Boxplots showing tear film cytokine levels in SARS-CoV-2-positive patients and SARS-CoV-2-negative controls. *p* values are written above the boxplots.

**Figure 2:**
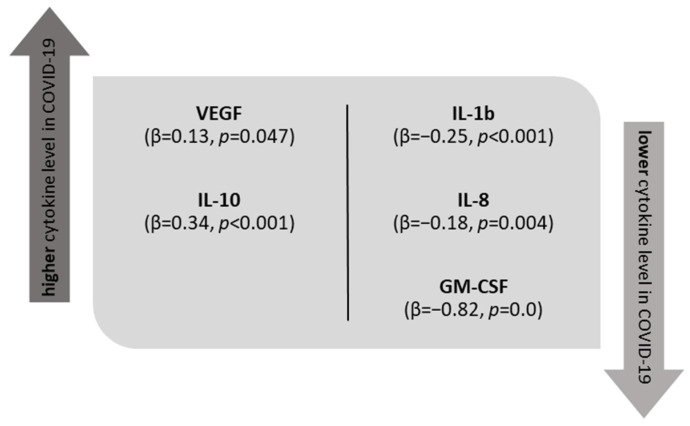
Graph showing the statistically significant results of the multivariate analysis adjusted for age and sex regarding the influence of COVID-19 on the cytokine levels.

The tear film concentrations of VEGF and IL-10 were found to be significantly increased, whereas TNF-α, IL-1β, IL-8, and GM-CSF were significantly lower among the COVID-19 patients admitted to the hospital than among the SARS-CoV-2-negative controls (Figure 1, Supplementary Figure S1 and Table 3). Figure 2 shows the statistically significant results of a multivariate analysis of patients and controls adjusted for age and sex. The analysis revealed that COVID-19 was an independent factor associated with higher VEGF and IL-10 levels (β = 0.13, *p* = 0.047, and β = 0.34, *p* < 0.001, respectively) and lower IL-1β, IL-8, and GM-CSF levels (β = −0.25, *p* < 0.001; β = −0.18, *p* = 0.004; and β = −0.82, *p* = 0.0, respectively).

## 4. Discussion

Our study was designed to evaluate the ophthalmologic characteristics of symptomatic COVID-19 patients admitted to the Infectious Diseases Department to obtain a complete ocular screening for SARS-CoV-2 infection. Several reports have described ocular surface manifestations during SARS-CoV-2 infection in variable percentages of patients [6,7,8,9,10,11]. As the precise incidence of the ocular manifestations of COVID-19 is still unclear, we analyzed the presence and duration of ophthalmic symptoms among a large number (340) of subjects with confirmed positive or negative RT–PCR results for SARS-CoV-2 genetic material in nasopharyngeal swabs. In the present study, we observed that the occurrence of ocular symptoms was different between the two tested groups, and that among those with positive results for SARS-CoV-2, 12.64% had at least one ECS, while 0.66% of the negative controls complained of at least one ECS. Our results were comparable with the rates established by other authors. Agrawal et al., in a meta-analysis that pooled data from 16 published studies on confirmed COVID-19 subjects, reported that 11.64% of COVID-19 patients had ocular surface manifestations [12]. Other studies found variable rates, ranging from 24% of patients with positive conjunctival symptoms [13] to 32% of COVID-19 subjects with ocular signs and symptoms [14].

Our research demonstrates that symptomatic SARS-CoV-2-positive patients reported a higher prevalence of eye redness, pain, itching burning, or tearing, all of which are symptoms that are highly indicative of the occurrence of conjunctival congestion with subsequent inflammatory reactions. Our findings are similar to those described by Hang et al. and Wu et al., who separately reported equal percentages of ocular signs, including redness, dryness, ocular pain, foreign body sensation, discharge, itching, and follicular conjunctivitis [1,5]. Xu et al. reported itching as the prime ocular symptom, whereas Karimi et al. described foreign-body sensation as the most frequent symptom in COVID-19 patients [15,16]. Shaikh et al. reported conjunctival hyperemia (33.3%) to be the most frequent ocular manifestation in their examined COVID-19 population [17]. Interestingly, we found no differences between the compared SARS-CoV-2-positive and SARS-CoV-2-negative groups when analyzing the presence of allergic or infectious conjunctivitis and dry eye within one year preceding the COVID-19 event. This finding indicates that the groups did not differ in terms of the risk factors predisposing them to infection complications of the eye surface. Generally, although the overall number of ocular symptoms in the COVID-19-infected population is low in most published studies, in one study, approximately 25% of COVID-19 patients with ophthalmological manifestations presented their ocular signs earlier than their other systemic manifestations, regardless of the severity of their COVID-19 infection [18]. These data are in line with the results of our study, indicating that ocular manifestations can occur early in the course of COVID-19. The ocular symptoms occurred within 7 days before admission to the hospital; this may indicate that conjunctival manifestations could also be preliminary signs of the development of COVID-19. It is worth mentioning that conjunctivitis and conjunctivitis-related symptoms may also be strongly underreported by different groups of patients with COVID-19, such as elderly or young people, given the nature of this eye disease, which is presumably self-limiting and rarely threatens vision.

Given the relevance of biomarker evaluation for disease monitoring and prognosis, tear sampling may be a non-invasive, easily accessible, and appropriate technique for the measurement of inflammatory cytokines in tears from COVID-19 patients. In our series of tear samples for inflammation-related cytokine measurement, we found that anti-inflammatory IL-10 was significantly increased, whereas potent proinflammatory molecules, such as TNF-α, IL-1β, and IL-8, were significantly lower in the tears from the COVID-19 patients than in those from the matched negative controls. The anti-inflammatory IL-10 naturally inhibits proinflammatory cytokine release and reduces conjunctival dendritic cell maturation; thus, it potentially blocks the complications of COVID-19 infection locally in the conjunctiva and ocular surface, which is directly exposed to contaminated droplets and fluid aerosols. Our results are in line with those of other studies concluding that coronaviruses do not seem to attract a strong humoral response from the conjunctival immune system and are rarely associated, therefore, with inflammatory reactions in ocular tissues [19]. By contrast, influenza or adenoviruses can often lead to conjunctivitis or keratitis [20] and it is well known that IL-8 may play a role in the development of subepithelial infiltrates in adenovirus keratitis [21]. Interestingly, IL-1β and IL-6 were documented to be significantly elevated in tears from bacteria-affected eyes [22]. Our study revealed that both IL-1 β and IL-8 were significantly lower in the COVID-19 patients. This indicates that different pathogens may stimulate specific pattern-recognition receptors on the cellular surface, leading to distinct immune responses. This may account for the infrequent presence of ocular symptoms in COVID-19 patients.

In summary, we revealed that COVID-19 does not induce local ocular hypercytokinemia, which might indicate that humoral immunity is not strongly facilitated on the ocular surface in the course of novel coronavirus infection. However, despite the low prevalence of ocular symptoms detected among all the patients infected by SARS-CoV-2, it is imperative for all ophthalmologists to understand the full spectrum of COVID-19-related signs and comorbidities. In particular, health care workers should pay attention to patients’ ocular manifestations in the early stages of COVID-19. These findings are meaningful due to the large number of subjects with COVID-19 worldwide and should be recognized by ophthalmologists as potential long-term sequalae of the disease.

## Figures and Tables

**Table 1:** Preoperative characteristics of the study groups—mean values ± SD. Statistically significant *p* values are shown in bold.

Parameter		SARS-CoV2-Negative Controls	SARS-CoV2-Positive Patients	*p*
Age (mean ± SD)		48.59 ± 9.60	50.32 ± 12.53	0.054
Sex (male/female)		17/143	102/78	**<0.001**
Body-mass index (mean ± SD)		26.17 ± 4.49	28.70 ± 5.66	**<0.001**
% of patients with a given parameter	Medical history:			
	Hypertension	13.16	32.78	**<0.001**
	Diabetes	1.97	12.78	**<0.001**
	Ischemic heart disease	0.00	3.33	**0.03**
	Hypercholesterolemia	5.92	8.89	0.40
	Liver disease	0.00	0.56	1.00
	Rheumatic disease	17.11	5.56	**0.001**
	Cancer	3.29	7.78	0.10
	Other diseases	0.00	27.22	**<0.001**
	Currently taken medications:			
	Statins	2.63	10.00	**0.01**
	NSAIDs	7.24	6.67	0.83
	Antihypertensive drugs	13.82	34.44	**<0.001**
	Anticoagulants	3.95	5.00	0.79
	Cardiac drugs	0.00	5.00	**0.004**
	Anti-asthmatic drugs	8.55	9.44	0.85
	Other drugs	23.68	33.33	0.07

**Table 2:** Ophthalmological characteristics of patients with positive and negative SARS-CoV-2 PCR results. Statistically significant *p* values are shown in bold.

	Ophthalmic Symptom	SARS-CoV2 Negative Controls	SARS-CoV2 Positive Patients	*p*
% of patients with a given ophthalmic symptom at the time of enrollment	Eyelids swelling	0.00	0.00	-
	Eye itching	0.66	1.15	1.00
	Eye burning	0.66	3.45	0.13
	Eye tearing	0.66	4.60	**0.04**
	Eye redness	0.00	2.30	0.13
	Sand sensation under the eyelid	0.66	0.57	1.00
	Presence of the discharge	0.00	1.72	0.25
	Gluing of the eyelids	0.00	1.15	0.50
	Light sensitivity	0.00	2.87	0.06
	Eye stifness	0.00	0.57	1.00
	Eye pain	0.00	4.60	**0.01**
	Visual impairment	0.66	0.57	1.00
	Misty vision	0.66	0.57	1.00
	Blurry vision	0.66	1.15	1.00
% of patients with a given ophthalmic symptom during the preceding 7 days	Eyelids swelling	0.66	1.15	1.00
	Eye itching	0.00	4.60	**0.01**
	Eye burning	0.66	5.75	**0.01**
	Eye tearing	0.66	8.05	**0.001**
	Eye redness	0.00	4.02	**0.02**
	Sandy sensation under the eyelid	0.66	2.87	0.22
	Presence of discharge	0.00	2.30	0.13
	Gluing of the eyelids	0.00	2.30	0.13
	Light sensitivity	0.66	4.60	**0.04**
	Eye stifness	0.00	2.30	0.13
	Eye pain	0.00	6.90	**<0.001**
	Visual impairment	0.00	3.45	**0.03**
	Misty vision	0.00	2.30	0.13
	Blurry vision	0.00	2.30	0.13

**Table 3:** Tear film cytokine levels among patients with positive and negative SARS-CoV-2 PCR results (pg/mL). Statistically significant values are shown in bold.

Parameter	SARS-CoV2 Negative Controls	SARS-CoV2 Positive Patients	*p*
	Mean ± SD	Mean ± SD	
TNF-α	1.27 ± 1.47	1.25 ± 2.94	**0.004**
VEGF	45.30 ± 25.87	53.42 ± 29.02	**0.01**
IL-2	0.32 ± 0.25	0.30 ± 0.22	0.74
IL-1β	1.94 ± 3.73	2.10 ± 8.90	**<0.001**
IL-4	15.39 ± 8.59	13.76 ± 5.60	0.08
IL-5	0.17 ± 0.17	0.15 ± 0.15	0.12
IL-6	0.82 ± 3.04	1.07 ± 4.59	0.77
IL-8	168.40 ± 537.92	80.79 ± 288.55	**<0.001**
IL-10	5.19 ± 3.68	8.77 ± 5.81	**<0.001**
IL-12	2.94 ± 11.35	2.25 ± 1.61	0.32
GM-CSF	1.13 ± 0.45	0.38 ± 0.48	**<0.001**
IFN-γ	1.30 ± 1.37	1.17 ± 0.83	0.33

## Data Availability

The data that were used to support the findings of this study are available from the corresponding author upon request.

## Supplementary Materials

The following supporting information can be downloaded at: https://www.mdpi.com/article/10.3390/jcm11092647/s1. Figure S1. Boxplots showing non-statistical tear film cytokine levels in SARS-CoV-2-positive patients and SARS-CoV-2-negative controls.

## References

[1] Wu P., Duan F., Luo C., Liu Q., Qu X., Liang L., Wu K. (2020). Characteristics of Ocular Findings of Patients With Coronavirus Disease 2019 (COVID-19) in Hubei Province, China. JAMA Ophthalmol..

[2] Rodríguez-Ares T., Lamas-Francis D., Treviño M., Navarro D., Cea M., López-Valladares M.J., Martínez L., Gude F., Touriño R. (2021). SARS-CoV-2 in Conjunctiva and Tears and Ocular Symptoms of Patients with COVID-19. Vision.

[3] Lu C.-W., Liu X.-F., Jia Z.-F. (2020). 2019-nCoV transmission through the ocular surface must not be ignored. Lancet.

[4] Xia J., Tong J., Liu M., Shen Y., Guo D. (2020). Evaluation of coronavirus in tears and conjunctival secretions of patients with SARS-CoV-2 infection. J. Med. Virol..

[5] Burgos-Blasco B., Güemes-Villahoz N., Santiago J.L., Fernandez-Vigo J.I., Espino-Paisán L., Sarriá B., García-Feijoo J., Martinez-De-La-Casa J.M. (2020). Hypercytokinemia in COVID-19: Tear cytokine profile in hospitalized COVID-19 patients. Exp. Eye Res..

[6] Petrillo F., Chianese A., De Bernardo M., Zannella C., Galdiero M., Reibaldi M., Avitabile T., Boccia G., Galdiero M., Rosa N. (2021). Inhibitory Effect of Ophthalmic Solutions against SARS-CoV-2: A Preventive Action to Block the Viral Transmission?. Microorganisms.

[7] Troisi M., Zannella C., Troisi S., De Bernardo M., Galdiero M., Franci G., Rosa N. (2022). Ocular Surface Infection by SARS-CoV-2 in COVID-19 Pneumonia Patients Admitted to Sub-Intensive Unit: Preliminary Results. Microorganisms.

[8] Zhou Y., Duan C., Zeng Y., Tong Y., Nie Y., Yang Y., Chen Z., Chen C. (2020). Ocular Findings and Proportion with Conjunctival SARS-COV-2 in COVID-19 Patients. Ophthalmology.

[9] Meduri A., Oliverio G.W., Mancuso G., Giuffrida A., Guarneri C., Rullo E.V., Nunnari G., Aragona P. (2020). Ocular surface manifestation of COVID-19 and tear film analysis. Sci. Rep..

[10] Mungmungpuntipantip R., Wiwanitkit V. (2020). Ocular manifestation, eye protection, and COVID-19. Graefes Arch. Clin. Exp. Ophthalmol..

[11] Hong N., Yu W., Xia J., Shen Y., Yap M., Han W. (2020). Evaluation of ocular symptoms and tropism of SARS-CoV-2 in patients confirmed with COVID-19. Acta Ophthalmol..

[12] Aggarwal K., Agarwal A., Jaiswal N., Dahiya N., Ahuja A., Mahajan S., Tong L., Duggal M., Singh M., Agrawal R. (2020). Ocular surface manifestations of coronavirus disease 2019 (COVID-19): A systematic review and meta-analysis. PLoS ONE.

[13] Arora R., Goel R., Kumar S., Chhabra M., Saxena S., Manchanda V., Pumma P. (2020). Evaluation of SARS-CoV-2 in Tears of Patients with Moderate to Severe COVID-19. Ophthalmology.

[14] Almazroa A., Alamri S., Alabdulkader B., Alkozi H., Khan A., Alghamdi W. (2021). Ocular transmission and manifestation for coronavirus disease: A systematic review. Int. Health.

[15] Xu L., Zhang X., Song W., Sun B., Mu J., Wang B., Wang Z., Cao Y., Dong X. (2020). Conjunctival Polymerase Chain Reaction-Tests of 2019 Novel Coronavirus in Patients in Shenyang, China (2/20/2020). China (2/20/2020).

[16] Karimi S., Arabi A., Shahraki T., Safi S. (2020). Detection of severe acute respiratory syndrome Coronavirus-2 in the tears of patients with Coronavirus disease 2019. Eye.

[17] Shaikh N., Al Mahdi H., Pai A., Pathare A., Abujaber A.A., Dsliva A., Al-Jabry M., Subramanian K., Thomas S., Mohmed A.S. (2022). Ocular manifestations of COVID-19: Facts and figures from a tertiary care center. Ann. Med..

[18] Nora R.L.D., Putera I., Khalisha D.F., Septiana I., Ridwan A.S., Sitompul R. (2020). Are eyes the windows to COVID-19? ystematic review and meta-analysis. SBMJ Open Ophthalmol..

[19] Belser J.A., Rota P.A., Tumpey T.M. (2013). Ocular Tropism of Respiratory Viruses. Microbiol. Mol. Biol. Rev..

[20] Creager H., Kumar A., Zeng H., Maines T.R., Tumpey T.M., Belser J.A. (2018). Infection and Replication of Influenza Virus at the Ocular Surface. J. Virol..

[21] Chodosh J., Astley R.A., Butler M.G., Kennedy R.C. (2000). Adenovirus keratitis: A role for interleukin-8. Investig. Ophthalmol. Vis. Sci..

[22] Yamaguchi T., Calvacanti B.M., Cruzat A., Qazi Y., Ishikawa S., Osuka A., Lederer J., Hamrah P. (2014). Correlation Between Human Tear Cytokine Levels and Cellular Corneal Changes in Patients With Bacterial Keratitis by In Vivo Confocal Microscopy. Investig. Opthalmol. Vis. Sci..

