# Supplementary material for: Evaluating Ocular Symptoms and Tear Film Cytokine Profiles in Symptomatic COVID-19 Patients
Source: J Clin Med. 2022 May 8;11(9):2647. doi: 10.3390/jcm11092647 (PMC9105717; doi:10.3390/jcm11092647)
Supplement: Supplementary file 1 [file jcm-11-02647-s001.zip › jcm-1705762-supplementary.pdf]

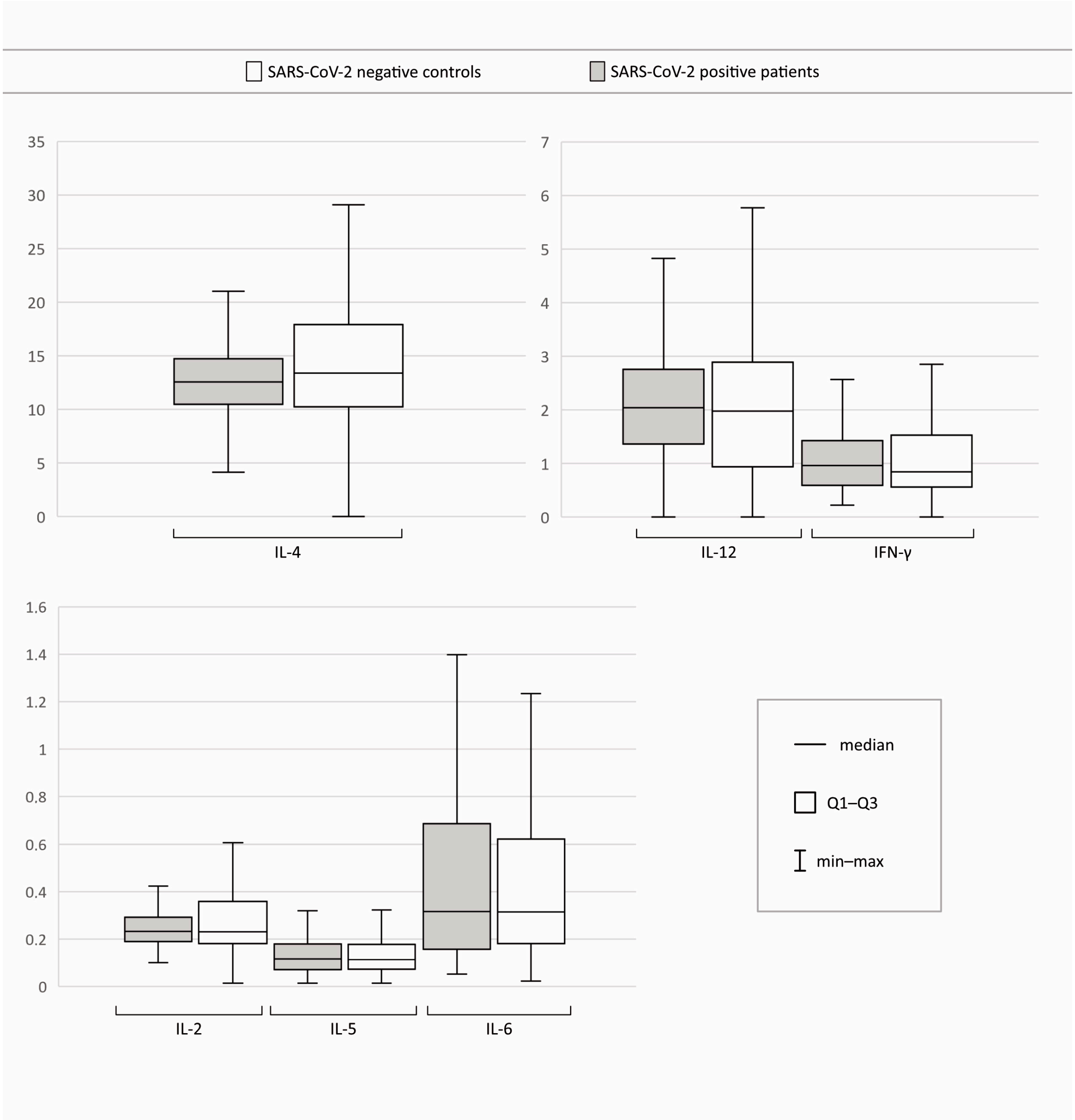

Figure S1. Boxplots showing non-statistical tear film cytokine levels in SARS-CoV-2-positive patients and SARS-CoV-2-negative controls.
